# The Hallmarks of Cancer as Ecologically Driven Phenotypes

**DOI:** 10.3389/fevo.2021.661583

**Published:** 2021-04-28

**Authors:** Jason A. Somarelli

**Affiliations:** Department of Medicine, Duke Cancer institute, Duke University Medical Center, Durham, NC, United States

**Keywords:** ideal free distribution, metastasis, tumor microenvironment, fitness, niche construction theory

## Abstract

Ecological fitness is the ability of individuals in a population to survive and reproduce. Individuals with increased fitness are better equipped to withstand the selective pressures of their environments. This paradigm pertains to all organismal life as we know it; however, it is also becoming increasingly clear that within multicellular organisms exist highly complex, competitive, and cooperative populations of cells under many of the same ecological and evolutionary constraints as populations of individuals in nature. In this review I discuss the parallels between populations of cancer cells and populations of individuals in the wild, highlighting how individuals in either context are constrained by their environments to converge on a small number of critical phenotypes to ensure survival and future reproductive success. I argue that the hallmarks of cancer can be distilled into key phenotypes necessary for cancer cell fitness: survival and reproduction. I posit that for therapeutic strategies to be maximally beneficial, they should seek to subvert these ecologically driven phenotypic responses.

## THE HALLMARKS OF CANCER AS ECOLOGICAL FITNESS PARAMETERS

**FIGURE 1 |:**
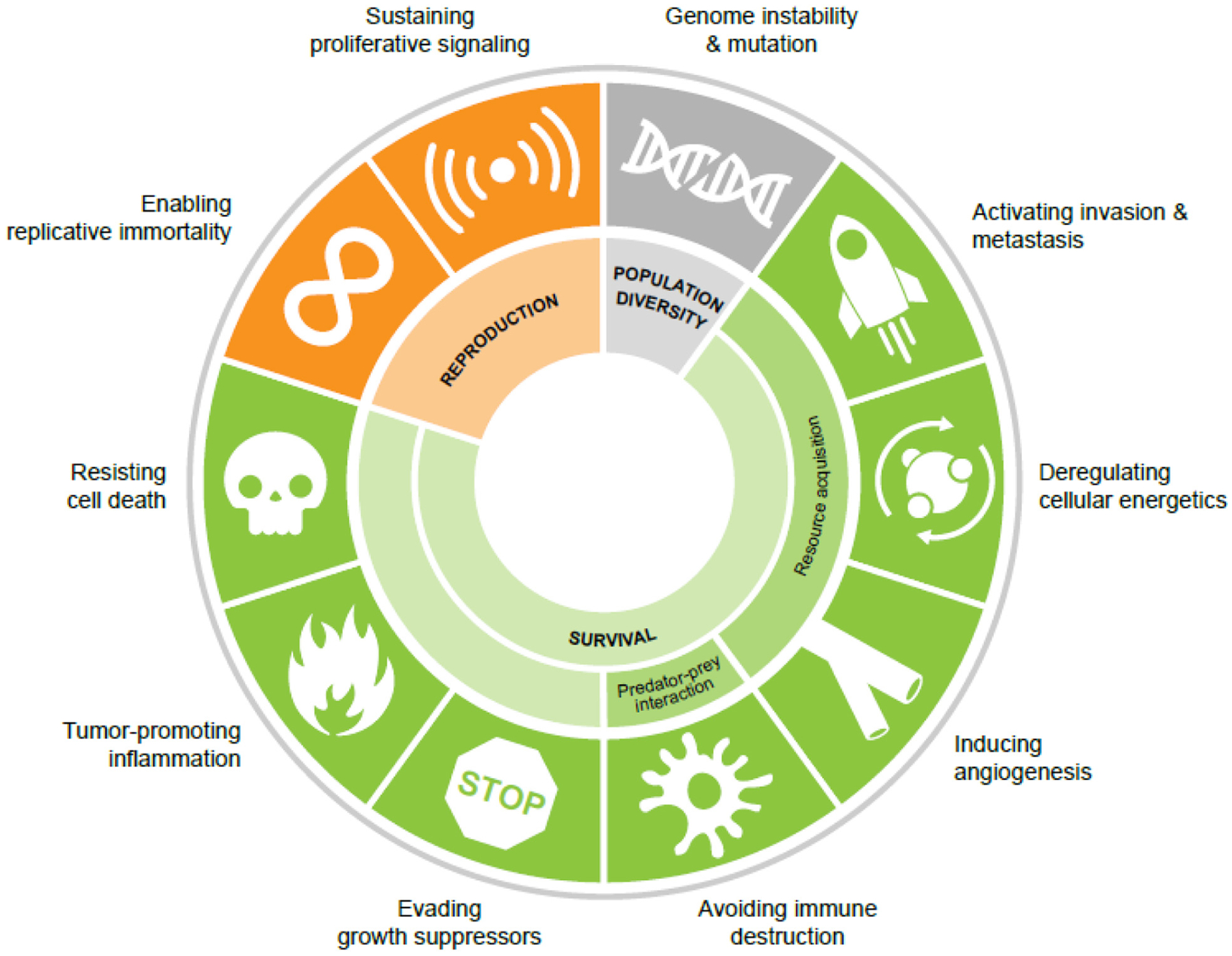
The cancer hallmarks as ecological fitness parameters. Population diversity is driven by genome instability and mutation. Cancer cell fitness is governed by a series of survival phenotypes and the ability to reproduce (proliferation and replicative immortality).

Cancer is a breakdown in multicellularity that is driven by genetic mutation, leading ultimately to unchecked growth (Aktipis et al., 2015). This unchecked growth of populations of monoclonally derived cells, coupled with continued genetic instability/mutation, epigenetic dysregulation, and stochastic variation in gene expression and post-transcriptional regulation, often creates a genotypically- and phenotypically-diverse population of cancer cells. In the context of solid tumors, this diverse population of cancer cells resides within a complex and dynamic ecosystem that is spatially distinct in its inhabitants, resources, and geography. Cancer cells must interact with this ecosystem to ensure their survival [reviewed in Somarelli et al. (2020)]. The phenotypic traits necessary for the continued presence of cancer in the body are known as the *cancer hallmarks*, which were eloquently described in two landmark papers by Hanahan and Weinberg (Hanahan and Weinberg, 2011; Figure 1).

Interestingly, while these hallmark phenotypes are observed across all cancers, the underlying genetic/epigenetic mechanisms that drive these phenotypes are remarkably heterogeneous. Indeed, efforts by The Cancer Genome Atlas^1^ and other consortia^2,3,4^ to genomically characterize multiple cancer types have illuminated this tremendous genetic and non-genetic diversity. The convergence of genotypically diverse individuals on a few key phenotypic traits is observed in ecological systems in the convergent evolution of phenotypes from genetically distinct species (Gatenby et al., 2011; Fortunato et al., 2017). Classic examples of this convergent evolution include the evolution of flight in insects, birds, and mammals (Chin and Lentink, 2016), the loss of sight and pigment in cave-dwelling fishes (Protas et al., 2006; Niven, 2008), and the evolution of fins and flippers in fishes and tetrapods (Fish and Lauder, 2017). Like these examples in nature, cancer cells, too, are constrained by their environments to converge on distinct phenotypic features that ensure their fitness within the ecology of the body. In this way, the cancer hallmarks represent the ecological fitness parameters of pro-survival and pro-reproduction (proliferation) phenotypes (Figure 1).

## CANCER CELLS EXIST WITHIN AN ECOLOGICAL SYSTEM

At its essence, what underlies the cancer hallmarks is an evolutionary fitness paradigm that describes key phenotypes necessary for survival and reproduction. In natural systems, the continued success of a species is defined by the fitness of its individuals. Fitness is the ability of an individual to survive and reproduce. At the population level, genetic and non-genetic variation within populations improves population-level fitness by increasing the likelihood that some individuals will survive and reproduce within a given ecological niche (Takahashi et al., 2018). An ecological niche includes all of the environmental conditions with which the individual interacts as well as the role played by the individual to shape its environment (Fath, 2018). Environmental conditions with which individuals interact include both biotic (e.g., predator/prey) and abiotic (e.g., geographic features) factors. These interactions dictate whether an individual maintains its fitness. Put simply, fitness is dependent upon a core set of phenotypes necessary for survival and successful reproduction. These core phenotypes can be achieved in myriad ways. For example, resource acquisition can be accomplished by altering food intake, migrating to new habitats/niches, or altering metabolism during periods of resource scarcity through hibernation or dormancy. Survival also includes diverse predatory escape/avoidance tactics.

**FIGURE 2 |:**
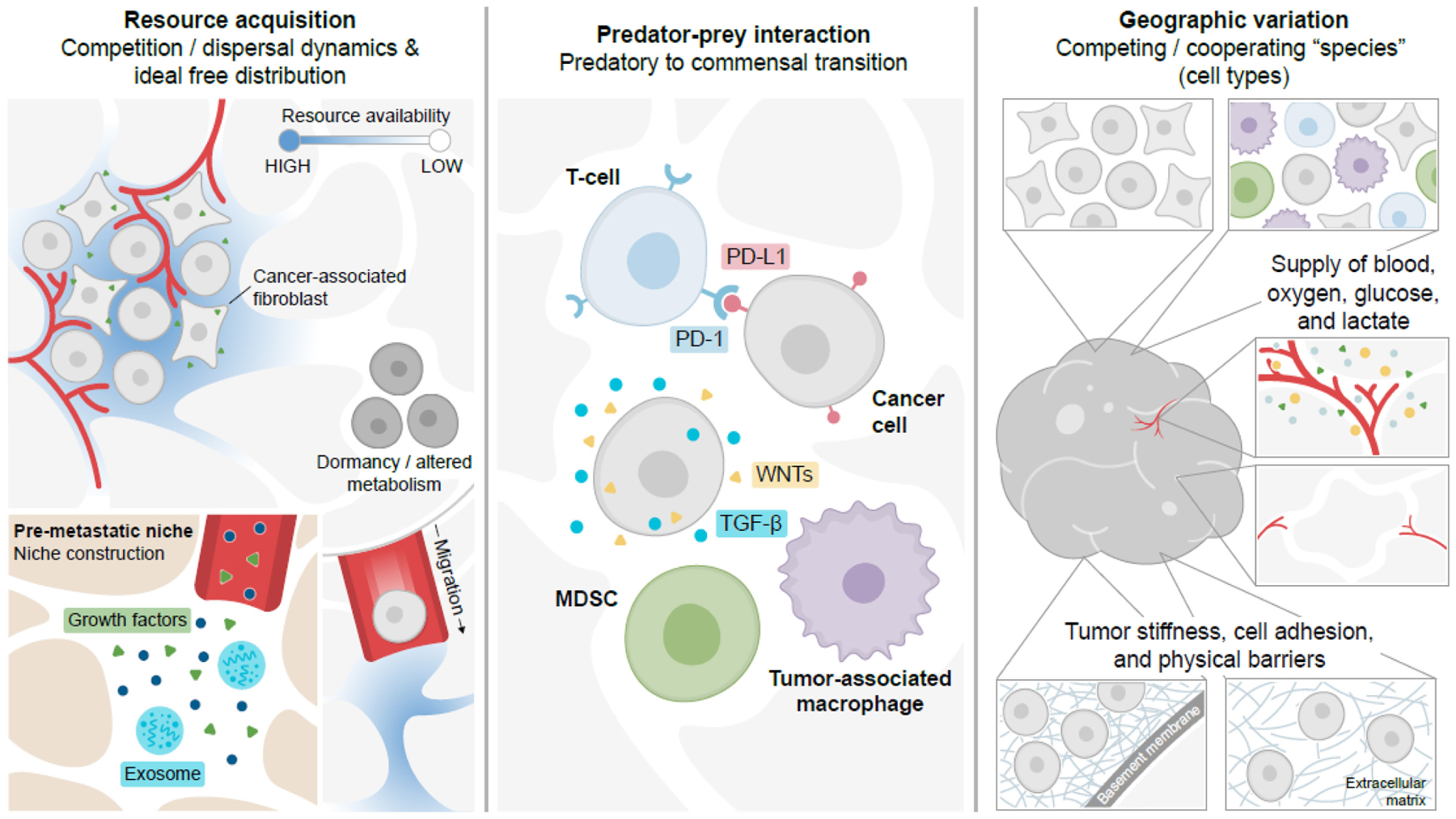
Cancer cell survival within the dynamic ecology of the body is driven by resource acquisition, predator-prey interactions, and geography. Cancer cells within solid tumors maintain their survival through alterations in resource acquisition (cell-intrinsic and -extrinsic signals, dormancy, migration/dispersal) and niche construction. Cells must also avoid predation by the immune system through immune evasive and immune suppressive responses. These parameters are shaped by geography and resource distributions within the tumor or metastatic landscape. MDSC, myeloid-derived suppressor cell; PD-L1 is an immune evasive checkpoint molecule; WNTs and TGF-β are soluble immunomodulatory signals.

Just as individual fitness is governed by interactions between individuals and their environments, cancers are also dependent upon the same core fitness phenotypes. With few exceptions (Andreoiu and Cheng, 2010), the vast majority of cancers originate from mutations within a single cell. Continued mutation of this clone during subsequent cell divisions leads to genetic diversity within a growing population of cancer cells. This genetic diversity is acted upon by selection for individual cells that can survive a specific ecological niche. Within solid tumors, the environment is spatially and temporally varied in the same way any natural environment would be, with multiple species co-existing within a dynamic, spatially diverse landscape (Figure 2). Like the natural world, the tumor is not merely a homogeneous cluster of cancer cells. Rather, the tumor is a pseudo-organ, comprised of both cancer and non-cancer cells co-existing together (Figure 2). These non-cancer cells, such as fibroblasts and other stromal cells (Sahai et al., 2020), endothelial cells (Hida et al., 2018), nerve cells (Banh et al., 2020), and immune cells (Binnewies et al., 2018) – which are often dysfunctional – contribute substantially to the tumor ecology by altering the resources and spatial geography of the tumor. In addition to the cells themselves, the local geography of the tumor is determined by vasculature, extracellular matrix components, resource availability, and tissue boundaries (Figure 2). This complex tumor environment shapes the survival phenotypes of resource availability and predation as well as the reproduction phenotype (Figure 2).

## THE ECOLOGY OF THE TUMOR SELECTS FOR CELLS THAT CAN SUCCESSFULLY FORAGE, AVOID PREDATION, MIGRATE, AND REPRODUCE

Resources, such as pro-survival signals, oxygen, and glucose are non-uniformly distributed throughout the tumor environment by the geography of the landscape and its non-cancer inhabitants (Milosevic et al., 1999; Rijken et al., 2000; Heaster et al., 2019; Zaidi et al., 2019). Neovascularization signals create a new blood supply that provides cancer cells with the oxygen, glucose, and growth factors that the cancer cells need for survival and reproduction. In addition to spatial heterogeneity in vasculature, resource distribution is also governed by the presence of non-cancer cells, many of which secrete signals in the form of growth factors, signaling ligands, or deposition of extracellular matrix components. Resource depletion can induce migratory/invasive properties (Yang et al., 2008; Chen et al., 2011; De Saedeleer et al., 2014). This relationship between resource depletion and migration is akin to the ecological concept of the ideal free distribution in which individuals within a population redistribute in a given environment to equalize resource intake rates (Fretwell and Lucas, 1969). While the ideal free distribution concept is most often studied in the context of vertebrate animal behavior, this concept also applies across species – from invertebrates (Kelly and Thompson, 2000) to single-celled organisms (Moses et al., 2013) – in response to the resource distributions within ecosystems. A deeper understanding of how this ecological concept can be applied to solid tumor biology may help identify new treatments to inhibit metastasis by shifting tumor ecology toward an environment that inhibits pro-migratory phenotypes. For example, spatio-temporal knowledge of the resource limitations and carrying capacity of the tumor environment may improve timing of intermittent therapies to inhibit migration/invasion programs in response to resource depletion or maintain drug sensitivity. Consistent with this notion, monitoring of spatial tumor hypoxia is being applied to adaptive radiation therapy strategies (Gerard et al., 2019), and monitoring the timing of metabolic reprogramming during therapy has been used to define targeted vulnerabilities to prolong treatment response in preclinical models of breast cancer (Goldman et al., 2019).

Ecological systems are shaped not only by resource distribution, but also by the predatory-prey interactions within the environment (Friman et al., 2008). Predator-prey relationships have profound consequences for evolutionary fitness. Predators can influence fitness of their prey by inducing physiological, morphological, or behavioral responses (Schmitz, 2017) and by inducing evolutionary selective forces on the prey population (Schmitz and Trussell, 2016). While cancer cells cannot exhibit behavioral changes *per se*, the profound influence of predators on population structure occurs not only in ecological contexts, but also in cancer. For example, cytotoxic T cells shift the ecological balance toward cancer cell prey that are able to thwart this predatory-like behavior of the immune system. Cancer cells escape immune predation through (1) increased expression of checkpoint molecules that enhance cancer cell tolerance (Pardoll, 2012) and (2) secretion of immunosuppressive factors that alter the phenotype of immune cells (Ben-Baruch, 2006). The factors produced by the cancer cells shift the relationship between cancer cell and immune cell from a predator-prey to a commensal interaction in which the cancer cell benefits from the newly established relationship by surviving.

The fitness parameter of reproduction in the context of cancer is proliferation by way of mitotic cell division. To divide, a cancer cell first needs to survive. However, while survival is a pre-requisite for this reproductive cell division, additional signals are also necessary to ensure reproductive success; as in nature, survival alone does not guarantee reproduction (i.e., cell division). Indeed, disseminated cancer cells have been shown to remain undetectable for decades (Recasens and Munoz, 2019; Shen et al., 2020). The reasons for the lack of clinical detection are numerous, including a technical limit on detection (Hori and Gambhir, 2011), activation of cellular pathways related to dormancy and hibernation (Klein, 2020), immune surveillance (Swann and Smyth, 2007), and growth constraint due to limited resources [reviewed in Klein (2011)]. In some cases, however, a subset of disseminated cancer cells can reawaken their proliferative capacity and cause a clinically detectable relapse. This reawakening can be promoted by a change in environment. For instance, resource depletion within the tumor, such as hypoxia, lactate production, reactive oxygen species, or the presence of inflammatory cytokines can lead to p38/MAPK-mediated stress signaling (Kyriakis and Avruch, 1996). The p38/MAPK pathway is intimately connected to cell cycle arrest (Takenaka et al., 1998). Interestingly, p38 activation also promotes cellular migration (Hamanoue et al., 2016), which may enable dormant cancer cells to escape resource depletion in the primary tumor for the more resource-rich environment of the metastatic niche. This trade-off between proliferation and migration is analogous to the competition/dispersal trade-off observed in ecological contexts in which habitat stability (Pellissier, 2015), population density (Matthysen, 2005), and carrying capacity (Laroche et al., 2016) affect dispersal dynamics, with higher density, lower resource availability, and lower carrying capacity promoting dispersal. Integrating these parameters of tumor ecology into models of cancer metastasis may improve our understanding of the (1) timing of metastasis and (2) clonal heterogeneity expected in a given patient. Advances in genomic profiling of liquid biopsies (Gupta et al., 2017, 2020; Armstrong et al., 2019; Ignatiadis et al., 2021) provide a powerful system to monitor competition/dispersal tradeoffs longitudinally and adjust treatment to minimize dispersal. This competition/dispersal theory has also illustrated how genetically- and phenotypically-similar species can co-exist within an ecological niche (Yawata et al., 2014). Applying these models to cancer may provide insight into the cancer cell phenotypes that may be most likely to co-exist within tumors and could help identify rational treatment combinations.

The switch from stress signaling in the primary tumor to a more favorable environment in a metastatic site may induce reawakening of proliferative signals through a shift in the ratio of activated, phosphorylated (phospho) ERK:phospho-p38 signaling (Aguirre-Ghiso et al., 2003). For example, reduction in TGF-β signaling (Bragado et al., 2013) and urokinase plasminogen activator signaling (Aguirre-Ghiso et al., 2003) in metastatic sites leads to a decrease in phospho-p38 levels and increase in phospho-ERK. Remarkably, the balance between phospho-ERK-mediated proliferation/reproduction and phospho-p38-mediated cell cycle arrest/dormancy in cancer cells is also observed in hibernating animals. Cardiac muscle from hibernating thirteen-lined ground squirrels (*Ictidomys tridecemlineatus*) exhibits a significant upregulation in phospho-p38 during torpor and a low phospho-ERK: phospho-p38 ratio (Childers et al., 2019). Likewise, skeletal muscle samples from hibernating bats display a significant increase in phospho-p38 (Eddy and Storey, 2007). Hibernation is an adaptation that trades immediate reproduction under resource scarcity for a later chance at reproduction in times of greater resource availability (Willis, 2017). In the same way, cancer cell dormancy is a fitness tradeoff that limits immediate reproduction to ensure survival in response to resource depletion.

## CANCER CELLS AND NICHE CONSTRUCTION

An ecological niche is the interaction between an organism and its environment. While this interaction is most often discussed from the perspective of the influence of the environment on the organism, the concept of a niche also includes the influence of the organism on its environment. The ability of the organism to re-shape its environment to create a more optimal niche is referred to as niche construction (Laland et al., 2016). This concept of niche construction is defined by the following properties: (1) the organism significantly modifies its environment, and (2) these environmental modifications impact the selection pressures of the organism (Odling-Smee et al., 2013). For example, dam building by beavers dramatically alters the landscape, creating ponds and lakes where streams once were. This alteration in the landscape not only creates new habitat for the beavers and other species, but it also provides a selective force on beaver traits, such as their social behaviors and disease vulnerabilities (Naiman et al., 1988). Notably, this selective force outlasts the beavers who built the dam, providing a selective advantage beyond the current generation.

Like beavers, cancer cells also substantially modify their environments in my, and in doing so influence their selection. For instance, tumor cells alter the geography of their environments through deposition and proteolytic cleavage and clearance of extracellular matrix components (Winkler et al., 2020). These proteolytic enzymes, such as matrix metalloprotease 2 and 9, are prognostic for poorer clinical outcomes in several cancer types (Grignon et al., 1996; Sier et al., 1996; Li et al., 2017; Huang, 2018). Mechanistically, these proteases alter matrix stiffness (Das et al., 2017), facilitate migration by creating space (Krause and Wolf, 2015), and increase pro-survival signaling (Augoff et al., 2020). In addition to remodeling their geography, cancer cells also remodel their nutrient sources. For example, in this issue, Wu et al. describe the process whereby tumors accumulate high concentrations of proliferation-limiting resources. Likewise, secretion of pro-angiogenic factors, such as vascular endothelial growth factor, fibroblast growth factors, epidermal growth factor, and platelet-derived growth factor, mediates formation of new vasculature (Bergers and Benjamin, 2003). In addition, tumor cells exert pressure on other cell types within the habitat of the tumor. Release of soluble factors by cancer cells can induce fibroblasts to switch from tumor suppressing to tumor permissive [reviewed in Alkasalias et al. (2018)]. Cancer cells can also signal to the immune predators in the tumor through expression of immune checkpoints on the cancer cells, such as PD-L1 and CTLA4 (Pardoll, 2012), or through secretion of soluble immunosuppressive factors, such as TGF-β (Wojtowicz-Praga, 2003), IL-10 (Kim et al., 2006), and soluble WNTs (Liang et al., 2014; Sun et al., 2017). This communication between cancer cells and immune subsets can lead to a restructuring of the immune landscape within the tumor toward a more tumor-tolerant environment.

Niche construction is not restricted to the local environment of the tumor. Systemic dissemination of signals also primes the pre-metastatic niche toward a cancer cell-permissive environment (Peinado et al., 2017; Doglioni et al., 2019). Signaling factors secreted by cancer cells can remodel distant sites for successful metastasis (Psaila and Lyden, 2009). The production of these secreted factors is also influenced by the local environment, thereby connecting local resource depletion and cell-to-cell crosstalk with distant niche construction in pre-metastatic organs. In mouse models of breast cancer, for example, tumor hypoxia led to the expression of lysyl oxidase, which induced recruitment of CD11b+ myeloid cells to remodel the collagen matrix of pre-metastatic lungs (Erler et al., 2009). Similarly, in preclinical models of lung adenocarcinoma and melanoma metastasis, conditioned media from tumor cells increased secretion of fibronectin in the pre-metastatic niche, which facilitated recruitment of tumor cell-promoting bone marrow-derived cells (Kaplan et al., 2005). Similar to secreted growth factors from tumor cells, exosomes carrying cargo throughout the body facilitate tumor cell seeding at pre-metastatic sites. These exosomes can harbor proteins (Costa-Silva et al., 2015; Hoshino et al., 2015), microRNAs (Rana et al., 2013; Zhou et al., 2014; Fong et al., 2015), and long non-coding RNAs, with impacts on the ecological niche, including extracellular matrix remodeling (Mu et al., 2013), angiogenesis and vascular permeability (Grange et al., 2011; Zeng et al., 2018), and immune cell populations (Liu et al., 2016; Wen et al., 2016). Unguided by an ecological perspective, many of the therapies that target these niche construction mechanisms have not been as successful as intended, and there is a remaining need to define the responses induced by cancer cells to remodel their niches, both locally and distally, at an individual patient level.

The most effective way to prevent the building of a dam is to remove the beavers before they cut down any trees. In the same way, early detection of cancer has been one of the most effective ways to improve cancer survival (Loud and Murphy, 2017). Despite their limitations, screening programs, particularly for colorectal, breast, cervical, prostate, skin, and other cancers have dramatically improved outcomes for cancer patients (Shieh et al., 2016; Loud and Murphy, 2017). While it has not been formally proven exactly how early detection has such a benefit, it is attractive to speculate that early removal of cancer cells prevents their (1) continued evolution toward more aggressive phenotypic states and (2) continued niche construction to create a permissive ecological landscape.

## THERAPY ALTERS THE CANCER CELL ECOLOGICAL NICHE, INDUCING RESPONSES THAT ARE BOTH BENEFICIAL AND DETRIMENTAL TO PATIENTS

**FIGURE 3 |:**
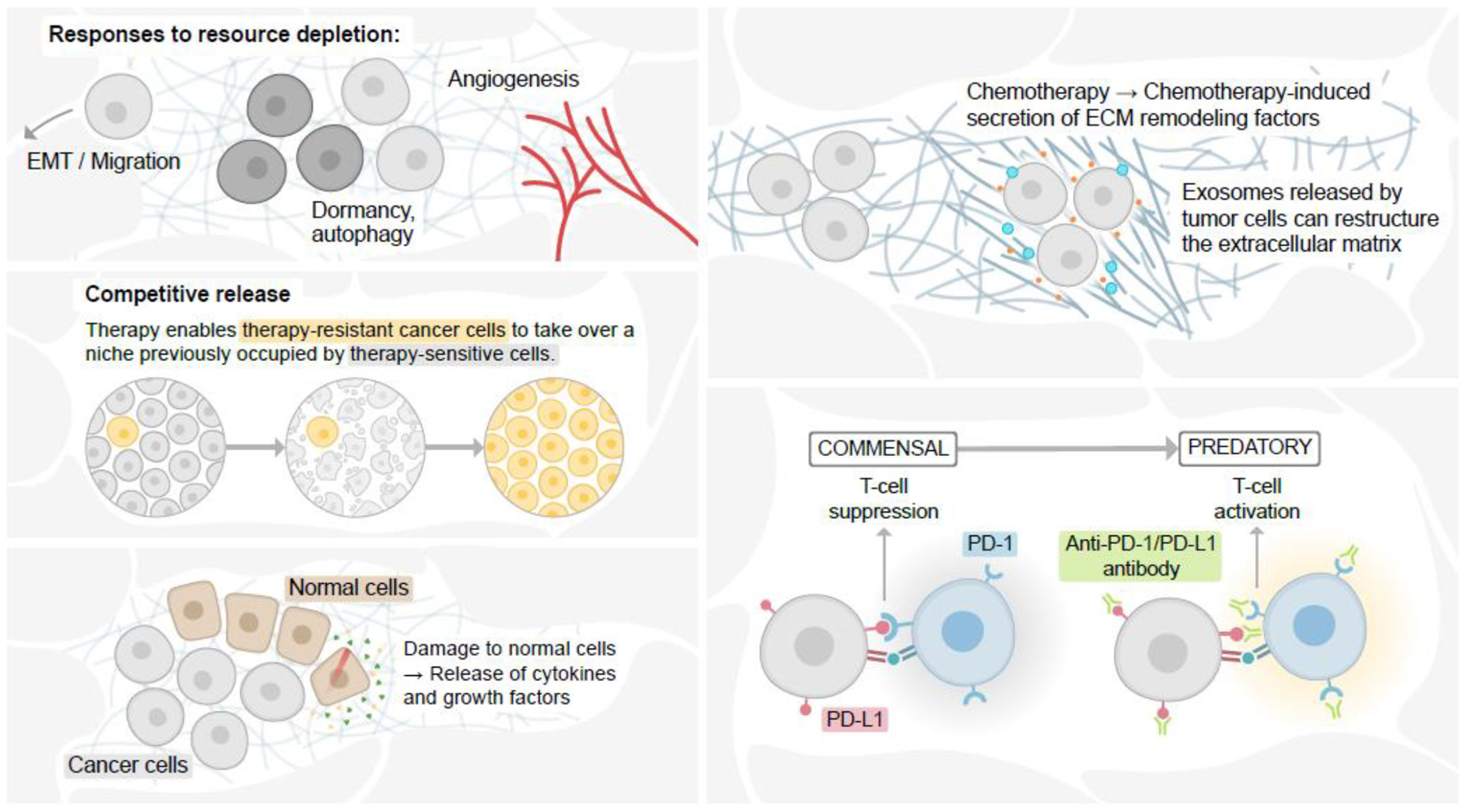
Therapy alters the cancer cell ecological niche. Cellular responses to resource depletion in the face of therapy can induce epithelial-mesenchymal transition (EMT) and migration, altered resource acquisition, and angiogenesis (**upper L**). Therapy-induced cellular damage can promote continued growth and tissue remodeling, providing growth factors and tissue space for remaining cancer cells to reoccupy the altered niche (**bottom L**). Removal of drug-sensitive cells from an ecological niche can allow drug-resistant cells to take over the new niche (**top R**). Immune checkpoint inhibition induces in the immune system a switch from a commensal to predatory.

Therapy substantially modifies the cancer cell population heterogeneity, fitness landscape, and ecology of the tumor (Figure 3). Whether by selection of a subclone with pre-existing resistance or the acquisition of a resistance mechanism in response to treatment, therapy often [though not always (Ding et al., 2012; Bashashati et al., 2013)] induces a strong selective bottleneck that enriches for resistant phenotypes within cancer cells. This can have profound impacts on population structure, as has been demonstrated in numerous cancer types through genomic profiling of longitudinal samples (Johnson et al., 2014; Gupta et al., 2017, 2020; Somarelli et al., 2017; Armstrong et al., 2019; Caswell-Jin et al., 2019; Roper et al., 2020). In addition, therapy-induced enrichment of resistant phenotypes can also promote additional aggressive features of cancer, such as altered resource acquisition (Lue et al., 2017; Xu et al., 2019; Gremke et al., 2020), dormancy (Kurppa et al., 2020; Ware et al., 2020), migration/invasion phenotypes (Takeuchi et al., 2015; Ware et al., 2016; Shah et al., 2017; Jolly et al., 2019), and immune evasion (Baghdadi et al., 2016; Ware et al., 2020).

In addition, the reshaping of the cancer cell population structure by therapeutic challenge can also alter the fitness landscape of the cell population in which removal of a drug-sensitive population allows drug-resistant cells to repopulate a newly vacant ecological niche (West et al., 2018; Figure 3). This ecological concept, known as competitive release, can be explained mechanistically by differences in energy expenditure within drug-sensitive and drug-resistant populations. In the case of cytotoxic chemotherapy, resistant cells expend substantial energy in response to the drug [reviewed in Silva et al. (2012) and Kam et al. (2014)]. This energy expenditure renders resistant cells less fit than sensitive cells. When the drug is removed, sensitive cells are able to outcompete the resistant cells for space within the newly available ecological niche.

While the goal of systemic therapy is to target the cancer cells, the therapy can also have unintended consequences on non-malignant cells within the ecological system, some of which can promote further aggressive features of the cancer cells. For example, treatment-induced damage to cells within the tumor microenvironment has been shown to release secreted factors that enhance cancer cell survival (Sun et al., 2012; Li et al., 2021). Likewise, chemotherapy can also remodel the surrounding geography of the extracellular matrix, leading to increased cancer cell survival (Bandari et al., 2018; Figure 3). Chemotherapy can also alter the immune landscape by damaging hematopoietic stem cell niches (Gardner, 1999), leading to immune suppression (Wu and Waxman, 2018). Chemotherapy has also been shown to suppress immune function through secretion of immunosuppressive factors, such as IL34 (Baghdadi et al., 2016) and granulocyte macrophage colony-stimulating factor (Takeuchi et al., 2015). Therapy-induced cancer cell phenotypic plasticity also induces a host of immunomodulatory signaling pathways (Alumkal et al., 2020). Unlike the mostly unintended effects of chemotherapy on the “species” of immune cells within the tumor and the body, however, immunotherapy is specifically designed to reprogram the interaction between cancer cells and the immune system from a commensal to a predatory relationship (Figure 3). Ongoing and future work aimed at modeling these interactions using ecological frameworks (Griffiths et al., 2020) could improve trial design, predictive and prognostic power, and identify new mechanisms or treatment strategies aimed at prolonging the lives of cancer patients.

## LEVERAGING ECOLOGICAL RESPONSES TO GUIDE NOVEL THERAPEUTIC STRATEGIES

Viewing cancer from an ecological perspective can impact treatment paradigms. For instance, adaptive therapy in which treatment doses and schedules are adjusted based on the differential fitness of resistant and sensitive populations in the context of a drug has significantly prolonged tumor control in preclinical models of breast and ovarian cancers (Enriquez-Navas et al., 2016), with ongoing clinical trials in prostate cancer (Zhang et al., 2017) and other cancers [discussed in Cunningham et al. (2020)]. While these strategies have the potential to provide novel concepts to control disease, it is also imperative that we have a clear understanding of the relative fitness differences across resistance mechanisms and in different contexts. Adaptive therapy regimens may need to take into consideration both the frequency and relative fitness of resistance genotype/phenotype relationships (Schaper and Louis, 2014). For example, the “arrival of the frequent” (Schaper and Louis, 2014) suggests that frequent, but less fit phenotypes can become fixed in a population while other, rare phenotypes exist with increased fitness.

While the predominant focus of adaptive therapy has been on differential fitness and competition within cancer cell populations, other benefits of adaptive therapy may exist that are related to the tumor microenvironment. Lower drug doses may prevent toxicity to immune predatory cells. In the context of dying tumor cells, reduced lymphopenia may improve systemic immune response to localized and disseminated cancer cells. Similarly, lower drug concentrations within the tumor microenvironment may reduce the induction of a migratory/invasive phenotype in response to drug-mediated resource depletion.

In addition to adaptive therapy, strategies to target the tumor microenvironment and alter tissue ecology could be leveraged for novel combinations to inhibit both cancer cell-intrinsic and cell-extrinsic survival signals (Jin and Jin, 2020), alter tissue structure/geography (Erler et al., 2009; Juarez et al., 2012), and enhance immune predation (Opzoomer et al., 2019). Another approach may be to capitalize on dormancy as a response to therapy-mediated resource-depletion. For example, by using sequential treatment paradigms to first force cells into persistence/dormancy-like phenotypes, and then target these persistent cells with a secondary agent (Cipponi et al., 2020; Shen et al., 2020), it may be possible to prolong survival for patients with therapy-resistant or micrometastatic disease.

## CONCLUSION

The range of possible genetic solutions available to cancer cells in order to ensure survival and proliferation is vast. These innumerable possible solutions are constrained by the ecology of the individual patient, as well as the fundamental needs for survival and proliferation under stress, resulting in a set of phenotypic hallmarks. The hallmarks, at their essence, represent the phenotypic *solutions* for maintaining fitness within the ecological niche of the body. Ecologically informed therapeutic strategies can take advantage of these phenotypic responses required for fitness by using novel treatment approaches. To do this, the landscape of fitness parameters for each patient should be defined in order to identify rationale combinations or targets that control multiple aspects of cancer cell fitness. Beyond genetic drivers alone, therapeutic strategies should also consider the following in defining the fitness landscape of each patient: (1) identifying key resources, (2) defining the reproduction vs. dormancy/survival axis for tumors, (3) characterizing population heterogeneity, (4) quantifying dispersal likelihood, and (5) defining the predator/commensal state of the immune system. Broader partnership between ecologists and cancer researchers/physicians will help inform these strategies and could lead to further breakthroughs and innovation that capitalize on advances in spatially resolved genomics, measurements of the temporal dynamics of cancer cell populations, and an emerging arsenal of therapies that target both cancer cells and their habitats. Coupling these emerging technologies with ecologically informed models of cancer may enhance our ability to treat cancer as a chronic, but controllable illness that will substantially prolong the lives of cancer patients.

## Footnotes

1
https://www.cancer.gov/about-nci/organization/ccg/research/structural-genomics/tcga

2
https://platform.stjude.cloud/

3
https://www.rarecancergenome.org/

4
https://depmap.org/portal/
